# High-Resolution Imaging of Morphological Changes Associated with Apoptosis and Necrosis Using Single-Cell Full-Field Optical Coherence Tomography

**DOI:** 10.3390/bios15080522

**Published:** 2025-08-09

**Authors:** Suyeon Kang, Kyeong Ryeol Kim, Minju Cho, Joonseup Hwang, Joon-Mo Yang, Jun Ki Kim, Woo June Choi

**Affiliations:** 1 Department of Convergence Medicine, Brain Korea 21 Project, University of Ulsan College of Medicine, Seoul 05505, Republic of Korea; tndusdl@mail.ulsan.ac.kr (S.K.); rudfuf7@mail.ulsan.ac.kr (K.R.K.); us00166@mail.ulsan.ac.kr (M.C.); iamjun96@mail.ulsan.ac.kr (J.H.); 2 Center for Photoacoustic Medical Instruments, Department of Biomedical Engineering, Ulsan National Institute of Science and Technology (UNIST), Ulsan 44919, Republic of Korea; jmyang@unist.ac.kr; 3 Biomedical Engineering Research Center, Asan Medical Center, Seoul 05505, Republic of Korea; 4 School of Electrical and Electronics Engineering, Chung-Ang University, Seoul 06974, Republic of Korea; 5 Department of Intelligent Semiconductor Engineering, Chung-Ang University, Seoul 06974, Republic of Korea

**Keywords:** full-field optical coherence tomography, apoptosis, necrosis, label-free, non-invasive, single-cell analysis, three-dimensional topographic mapping, interference reflection microscopy-like imaging, time-lapse imaging

## Abstract

Full-field optical coherence tomography (FF-OCT) is a high-resolution interferometric imaging technique that enables label-free visualization of cellular structural changes. In this study, we employed a custom-built time-domain FF-OCT system to monitor morphological alterations in HeLa cells undergoing doxorubicin-induced apoptosis and ethanol-induced necrosis at the single-cell level. Apoptotic cells showed characteristic features such as echinoid spine formation, cell contraction, membrane blebbing, and filopodia reorganization. In contrast, necrotic cells exhibited rapid membrane rupture, intracellular content leakage, and abrupt loss of adhesion structure. These dynamic events were visualized using high-resolution tomography and three-dimensional surface topography mapping. Furthermore, FF-OCT-based interference reflection microscopy (IRM)-like imaging effectively highlighted changes in cell–substrate adhesion and cell boundary integrity during the cell death process. Our findings suggest that FF-OCT is a powerful imaging platform for distinguishing cell death pathways and assessing dynamic cellular states, with potential applications in drug toxicity testing, anticancer therapy evaluation, and regenerative medicine.

## 1. Introduction

Cells are the fundamental units of life, and their timely death is essential for maintaining tissue homeostasis and organismal survival [1,2,3]. Apoptosis, a genetically regulated process, is triggered by intracellular signaling and is characterized by the silent removal of cells without eliciting an inflammatory response [4,5,6]. It participates in various physiological processes such as development, tissue remodeling, and immune regulation. In contrast, necrosis is an uncontrolled form of cell death caused by physicochemical injury, which leads to cell membrane rupture and the leakage of intracellular contents, subsequently triggering an inflammatory response in the surrounding tissues [7,8]. Necrosis predominantly occurs under pathological conditions such as trauma, infection, and ischemia, and is closely associated with tissue damage and disease progression [3]. Both apoptosis and necrosis have significant implications for human health and disease pathology, making the accurate distinction between these processes vital in biomedical fields such as pathological diagnostics, drug response assessment, and cancer treatment. However, due to the presence of some morphologically similar features, high-resolution imaging techniques are necessary for precise discrimination.

Various biological imaging techniques, such as fluorescence staining, flow cytometry, electron microscopy, and Raman spectroscopy, have been employed to distinguish apoptosis and necrosis by detecting biochemical indicators, including cell membrane permeability, DNA fragmentation, and changes in mitochondrial function [9,10,11,12]. However, these methods have several limitations. First, most so-called non-invasive techniques still require intracellular chemical or fluorescent staining, which can adversely affect cell viability or structural integrity. In particular, fluorescent staining often suffers from reduced reliability due to photobleaching and nonspecific binding. Second, high-resolution techniques such as electron microscopy allow for detailed observation of cellular microstructures but necessitate fixation and sectioning of samples, precluding real-time analysis and live cell monitoring. These methods are also time-consuming and costly, limiting their feasibility for large-scale studies. Third, conventional approaches generally lack the capacity to intuitively render the three-dimensional (3D) morphology of intracellular structures and often require highly specialized analytical skills. Therefore, there is a critical need for a label-free, non-invasive, high-resolution imaging method capable of enabling rapid and accurate morphology-based cell-state analysis.

Quantitative phase microscopy (QPM) enables high-resolution, label-free, non-invasive imaging of the morphological features of living cells in real time without fixation. This technique quantitatively measures phase shifts in transmitted light and visualizes cell status by mapping the density distribution and refractive index (RI) variations within intracellular structures [13]. Owing to these characteristics, QPM is considered suitable for distinguishing subtle structural differences between apoptotic and necrotic cells [14,15,16]. Recent studies have successfully differentiated between distinct stages of apoptosis by quantitatively analyzing changes in phase information. However, QPM has several limitations. First, when internal structural boundaries are indistinct or RI contrast is low, fine structural details may remain unresolved. This limitation arises because phase information extraction depends on RI differences, making imaging resolution and contrast largely contingent on the cell’s RI distribution. Second, accurate interpretation of phase images requires complex mathematical processing and calibration, which increases data processing time and reduces user convenience. Third, since QPM generally provides only two-dimensional (2D) phase maps, it has limitations in fully reconstructing the 3D structure of cells, and phase distortion may occur due to scattering or multiple reflections. Therefore, further research is needed to optimize the resolution, processing speed, and 3D restoration capabilities of QPM and related technologies.

In this study, we utilized full-field optical coherence tomography (FF-OCT) to visualize apoptotic and necrotic processes at the single-cell level. FF-OCT is a variant of ultrahigh-resolution OCT technologies, based on Linnik interferometer configuration that detects light signals scattered from living sample structures within the coherence gate [17,18]. Unlike conventional OCT, which relies on point-by-point laser scanning, FF-OCT simultaneously illuminates and detects the entire field of view, enabling rapid and scan-free area observations in *en face* view. The use of a broadband light source (e.g., halogen lamp) in combination with high magnification objective lenses achieves both high axial and transverse resolution simultaneously, allowing for subcellular 3D imaging. As a label-free, non-invasive imaging modality, FF-OCT can observe single-cell structures without inflicting damage on cells or tissues, making it particularly suited for analyzing 3D architectures and internal organelle distributions through vertical tomography [19,20,21,22]. Accordingly, FF-OCT represents an ideal platform for high-resolution visualization and monitoring of the morphological changes associated with apoptosis and necrosis.

## 2. Materials and Methods

### 2.1. Apoptotic and Necrotic Cell Preparation

HeLa cells, derived from human cervical cancer cells (KCLB-10002, Korean Cell Line Bank, Seoul, Republic of Korea), were cultured as a monolayer in 100 mm dishes containing Dulbecco’s Modified Eagle’s Medium (DMEM) under 5% CO_2_ at 37 °C. Cells were maintained by routine subculturing in the Cell Culture Core Facility of Asan Medical Center under Biosafety Level 2 (BSL-2) conditions. All *in vitro* experiments were conducted in accordance with the biosafety guidelines and standard operating procedures of Asan Medical Center. To induce apoptosis in experimental group 1, doxorubicin was added to the culture medium at a final concentration of 5 μmol/L in a total volume of 1.5 mL. Doxorubicin is an anthracycline chemotherapeutic agent that induces apoptosis in rapidly proliferating cancer cells by intercalating into cellular DNA or inhibiting DNA replication via Topoisomerase II inhibition, thereby causing double-strand breaks [23,24,25]. This process activates intracellular injury responses, such as the p53 pathway, leading to cell apoptosis, while simultaneously increasing reactive oxygen species (ROS), which further amplifies intracellular stress. To induce necrosis, experimental group 2 was treated with 99% ethanol under the same incubation conditions. Ethanol causes nonspecific and rapid cellular damage at high concentrations, inducing abnormal cell death (necrosis) [26,27,28]. Due to its lipophilic nature, ethanol penetrates the phospholipid bilayer, increasing membrane permeability or disrupting membrane integrity, which promotes cellular leakage. Additionally, ethanol denatures the tertiary structure of proteins, resulting in inactivation of intracellular enzymes and structural proteins. This disruption of protein function impairs ion and water homeostasis, ultimately leading to cell swelling and rupture. Drug treatments were applied independently to each group without a control group. FF-OCT imaging was initiated immediately after drug administration in both groups and was performed continuously at 20-min intervals to monitor and analyze morphological changes for up to 180 min.

### 2.2. FF-OCT System Description

We evaluated real-time morphological changes at each stage of the apoptotic and necrotic processes using a custom-built time-domain FF-OCT system [29]. A broadband halogen light source (OSL2, center wavelength: 650 nm, spectral width: 200 nm, Thorlabs, Newton, NJ, USA) was employed to achieve sub-micrometer axial resolution. A Linnik-configured Michelson interferometer was constructed with identical 40× water-immersion objectives (LUMPLFLN40XW, numerical aperture (NA): 0.8, working distance (WD): 3.3 mm, Olympus, Tokyo, Japan) in both the reference and sample arms to enable subcellular-resolution (<1 μm) symmetrical imaging. A precision linear stage was used to control the optical path length difference along the depth in the *z*-axis by positioning the coherence gate at a specific cellular depth. When the cell structure was located within the coherence gate, light reflected from the sample and reference arms interfered and was detected as a 2D interference image by the image sensors, specifically a CCD camera (CCD-1020, 1024 × 1024 pixels, 12 bits, 20 fps, VDS Vosskühler GmbH, Osnabrück, Germany). Phase shifting was implemented by rapidly oscillating a piezoelectric actuator (PAZ005, 20 nm resolution, Thorlabs, Newton, NJ, USA) attached to the reference mirror along the *z*-axis, enabling the sequential acquisition of interference images with successive phase shifts. These temporal phase-shifted images were arithmetically processed to remove the DC component, isolating the sample reflection information and generating an *en face* (*x*-*y*) cross-sectional image of the target area. The acquired tomographic images were stacked in a *z*-stack format using a precise motorized sample stage, and the volume and surface morphology of the cell structure were reconstructed and analyzed in three dimensions.

### 2.3. FF-OCT-Based 3D Topography

The continuous cross-sectional data obtained with the FF-OCT system enabled 3D visualization of cellular internal structures and quantitative morphological analysis of cell surfaces. The depth of maximum intensity, defined as the *z*-position at which the reflected intensity was greatest in each A-scan (pixel-level *z*-axis signal), was identified as the cell surface. These maximum intensity positions were mapped across all pixels in the 2D *xy*-plane to generate a 3D point cloud. Spline interpolation was then applied to the point cloud to reconstruct a smooth topographic surface, allowing for the detailed visualization of cell surface morphology. This reconstruction facilitated time-lapse morphometric measurements in single cell structure.

### 2.4. FF-OCT-Based Interference Reflection Microscopy (IRM) Imaging

Although the FF-OCT platform was originally designed for tomographic imaging, it can also generate *en face* 2D interference images, similar to interference reflection microscopy (IRM), by adjusting the coherence gate position [30,31]. When the coherence gate is aligned near the culture substrate (culture dish’s surface) or the bottom of cell, interference arises from reflections at the cell–substrate interface. In this configuration, the resulting patterns are highly sensitive to nanoscale variations in the cell–substrate distance, providing IRM-like contrast. This imaging mode enables visualization of focal adhesion dynamics and changes in the cell–substrate interface. Additionally, IRM-like FF-OCT imaging reveals reflective contrast patterns across the entire cell, which vary depending on morphological and compositional changes. These contrasts are analogous to those observed with QPM, though achieved through reflection rather than transmission. Thus, the FF-OCT system functions as a multimodal imaging platform that integrates features of IRM and QPM-like imaging with FF-OCT.

## 3. Results

### 3.1. FF-OCT Imaging of a Single HeLa Cell Undergoing Apoptosis

**Figure 1:**
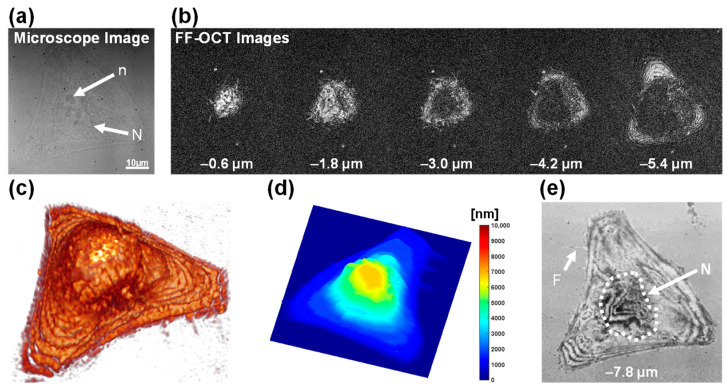
FF-OCT imaging of a single healthy HeLa cell in culture. (**a**) Bright-field microscopy image showing nucleus (N) and nucleolus (n); scale bar: 10 µm. (**b**) *En face* FF-OCT images acquired at five focal depths (–0.6 to –5.4 μm). (**c**) Volumetric rendering reconstructed from FF-OCT *z*-stack images. (**d**) Surface topography map based on peak-intensity depth information. (**e**) IRM-like image showing cell–substrate contacts and filopodia (F).

Figure 1 illustrates FF-OCT imaging of a single healthy HeLa cell in culture. In the bright-field microscope image of the cell (Figure 1a), the cell nucleus (*N*) and nucleolus (*n*) are visible, along with the overall translucent cellular morphology. Representative *en face* FF-OCT images (Figure 1b), acquired at five focal depths (−0.6 to −5.4 μm), provide depth-resolved tomograms of the HeLa cell. 3D reconstruction using the depth-resolved data yields a volumetric rendering of the cell shape and internal reflectivity distribution (Figure 1c). The surface topography map (Figure 1d), which color-codes the depth of the maximum reflection at each *xy* coordinate, reveals a dome-shaped elevation at the cell center with a gentle slope towards the periphery. IRM-like imaging was performed by positioning the coherence gate near the culture dish surface, and the result is shown in Figure 1e. This IRM-like image exhibits optical thickness contrast, highlighting filopodial microprotrusions (F) and characteristic interference patterns along the cell boundaries, indicative of cell–substrate gaps. These results demonstrate that FF-OCT is a versatile, high-resolution, label-free imaging platform, capable of simultaneously visualizing internal and external structures, 3D morphology, surface topography, and cell–matrix interactions at the single-cell level.

**Figure 2:**
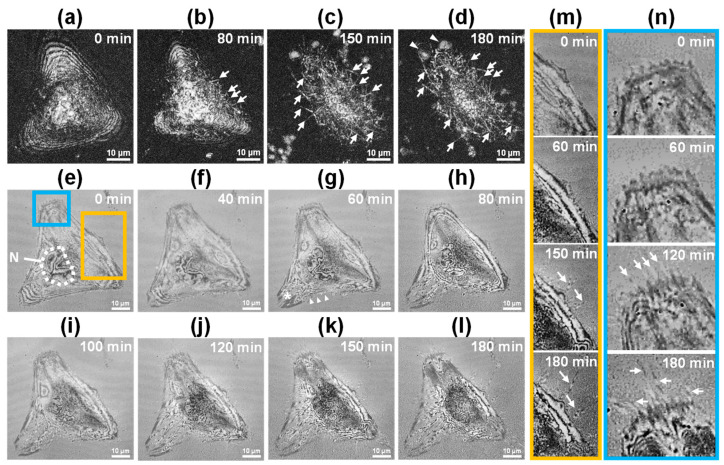
Time-lapse FF-OCT and IRM-like imaging of a single HeLa cell undergoing apoptosis after doxorubicin treatment. (**a**–**d**) Depth-integrated FF-OCT images at 0, 80, 150, and 180 min post-treatment, showing the formation of echinoid spikes (arrows) and blebs (arrowheads); scale bar: 10 µm. (**e**–**l**) IRM-like FF-OCT images of the cell–substrate region at multiple time points, highlighting membrane rupture (asterisk) and structural fragmentation (arrowheads in (**g**)); scale bar: 10 µm. (**m**,**n**) Magnified views of the yellow (**m**) and blue (**n**) boxed areas in (**e**), showing filopodial emergence, elongation, and dynamic reorganization of the cell–substrate interface.

Figure 2 illustrates the morphological changes in the HeLa cell over 180 min following doxorubicin treatment, as observed by FF-OCT and IRM-like imaging. FF-OCT cross-sectional imaging revealed a relatively homogeneous reflection pattern at 0 min after drug treatment (Figure 2a), with emergence of irregular protrusions (echinoid spikes) on the cell surface starting at 80 min (indicated by arrows in Figure 2b). These protrusions progressively increased in both density and size, spreading across the entire cell by 150 min (Figure 2c) and evolving into prominent bleb structures by 180 min (Figure 2d). IRM-like imaging (Figure 2e–l) highlighted the cell–substrate interface and revealed detailed structural breakdown characteristic of apoptosis. Between 40 and 60 min post-treatment (Figure 2f,g), the plasma membrane of the cell, which was in direct contact with the culture substrate, began to rupture (marked by an asterisk and arrowheads in Figure 2g), accompanied by fragmentation of internal structures, indicating the onset of early apoptosis. Between 120 and 180 min (Figure 2j–l), the cell exhibited progressive contraction, a decrease in the cell–substrate interference pattern, and eventual collapse of cellular architecture. In particular, we observed dynamic filopodial remodeling, characterized by elongation and increased filopodial abundance over time (arrows in yellow-boxed area in Figure 2m). A marked increase in filopodial density (arrows in blue-boxed region in Figure 2n) indicated active reorganization of cell–substrate adhesion. These results demonstrate that multimodal FF-OCT and IRM-like imaging enable comprehensive analysis of internal structural breakdown, membrane dynamics, and adhesion remodeling during apoptosis at the single-cell level.

### 3.2. FF-OCT-Based Topographic Mapping of the Apoptotic HeLa Cell

**Figure 3:**
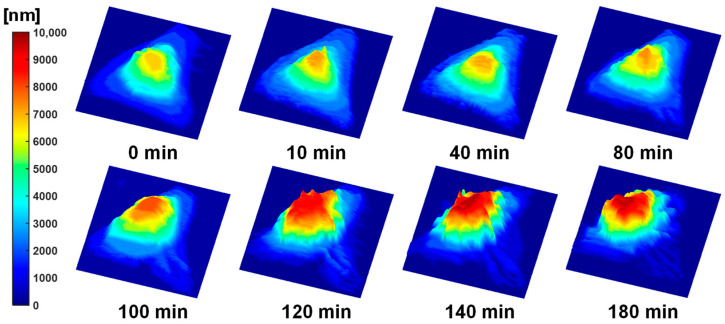
Time-lapse FF-OCT-based 3D topographic maps of an apoptotic HeLa cell following doxorubicin treatment. Surface height distributions at 0, 10, 40, 80, 100, 120, 140, and 180 min, color-coded in nanometers, showing progressive central protrusion and peripheral collapse.

Figure 3 shows the 3D surface topography of the apoptotic HeLa cell in Figure 2. In the pre-treatment map (0 min), the central region of the cell exhibited a uniform surface profile with a gentle dome-shaped protrusion measuring approximately 4–5 μm in height. No significant changes were observed at 10 and 40 min. Starting at 80 min, the central protrusion gradually increased in size, accompanied by a slight steepening of the slope. Pronounced morphological changes appeared at 100 min post-treatment, with the central protrusion rapidly increasing to 6–7 μm. This protrusion further expanded to 8–9 μm by 120 min and reached nearly 10 μm at 140 and 180 min. The observed central protrusion and peripheral depression were attributed to apoptotic volume decrease (AVD) and cytoskeletal stress redistribution during apoptosis. These findings indicate that FF-OCT-based topographic mapping facilitates morphological analysis, surpassing conventional phase imaging techniques by enabling the simultaneous, quantitative assessment of temporal and spatial changes in cell surface height.

### 3.3. FF-OCT Imaging of Single HeLa Cells Undergoing Necrosis

**Figure 4:**
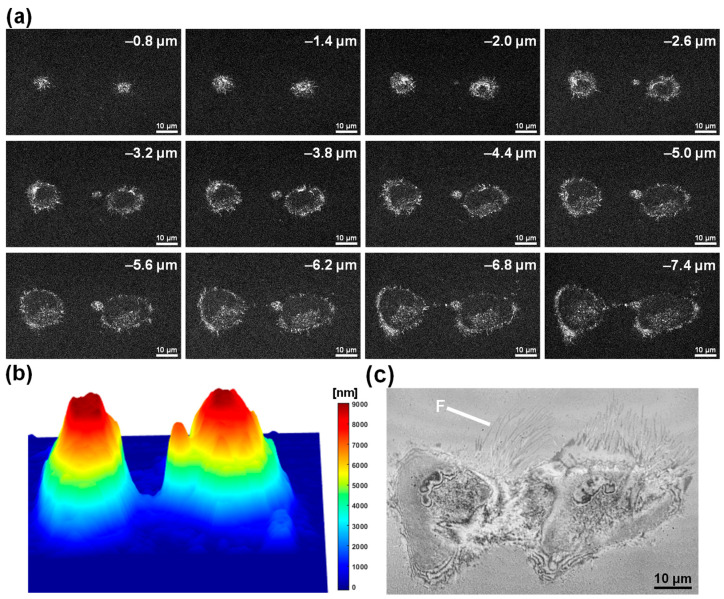
Baseline FF-OCT and IRM-like imaging of two healthy HeLa cells. (**a**) *En face* FF-OCT images at depths ranging from −0.8 to −7.4 μm, visualizing the nucleus and cytoplasmic boundaries; scale bar: 10 µm. (**b**) 3D topographic map showing surface height profiles, with maximum elevation observed near the nuclear region. (**c**) IRM-like imaging at the cell–substrate interface, highlighting filopodia (F) and cell–substrate adhesion patterns; scale bar: 10 µm.

FF-OCT imaging was performed on two healthy HeLa cells before ethanol-induced necrosis to record the structural and topographical characteristics of the normal state, with imaging depths ranging from −0.8 to −7.4 μm (Figure 4a). At each depth, the boundaries between the nucleus and cytoplasm were clearly visible, and the internal structures of the monolayer-adherent cells were well resolved. The 3D topographic maps, generated based on the peak reflection depth at each *xy* coordinate (Figure 4b), revealed a dome-shaped elevation of approximately 8–9 μm in the nuclear region of both cells, representing typical healthy cell morphology. IRM-like imaging (Figure 4c) shows fine filopodial projections (F) extending from the plasma membrane of the cell, along with cell–substrate interference patterns, indicating strong cell adhesion to the culture dish surface. These baseline FF-OCT and IRM-like images provide reference data for direct comparison with necrotic features, such as structural collapse, reductions in surface height, and filopodial loss, following ethanol treatment.

**Figure 5:**
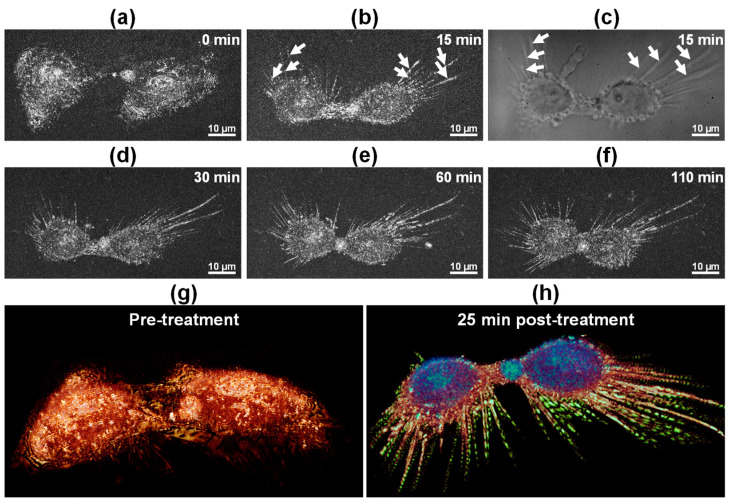
Time-lapse FF-OCT imaging of ethanol-induced necrosis in HeLa cells. (**a**) FF-OCT image at 0 min (pre-treatment). (**b**) FF-OCT image at 15 min, showing early exudation (arrows); scale bar: 10 µm. (**c**) Bright-field image at 15 min confirming cellular leakage; scale bar = 10 µm. (**d**–**f**) FF-OCT images at 30, 60, and 110 min, illustrating progressive cell deformation and exudate dispersal; scale bar: 10 µm. (**g**,**h**) 3D FF-OCT renderings before (**g**) and 25 min after (**h**) treatment, showing segmented structures: cell bodies (blue), exudates (yellow), and filopodia (green).

Figure 5 shows the time-lapse imaging of ethanol-induced necrosis using FF-OCT and volume rendering. Immediately prior to treatment, FF-OCT images revealed well-defined boundaries between the two cells and visible internal reflective structures, with cells maintaining stable morphology (Figure 5a). However, by 15 min post-treatment, strong scattering signals were observed emanating from the cells (arrows in Figure 5b), indicative of cellular exudation resulting from membrane rupture. At the same time point, bright-field imaging (Figure 5c) confirmed cellular leakage, demonstrating a strong correlation between FF-OCT signals and exudate structures. At 30, 60, and 110 min, cross-sectional FF-OCT images (Figure 5d–f) displayed progressive membrane damage, deformation of cell bodies, and loss of reflectivity. Volume rendering, obtained before treatment and 25 min after treatment, illustrated structural collapse and material dispersion during necrosis (Figure 5g,h). These findings demonstrate that FF-OCT enables real-time, label-free, non-invasive imaging of necrotic processes at the single-cell level, providing precise 3D visualization of material leakage and extracellular structural damage.

### 3.4. FF-OCT-Based IRM-like Imaging of the Necrotic HeLa Cells

**Figure 6:**
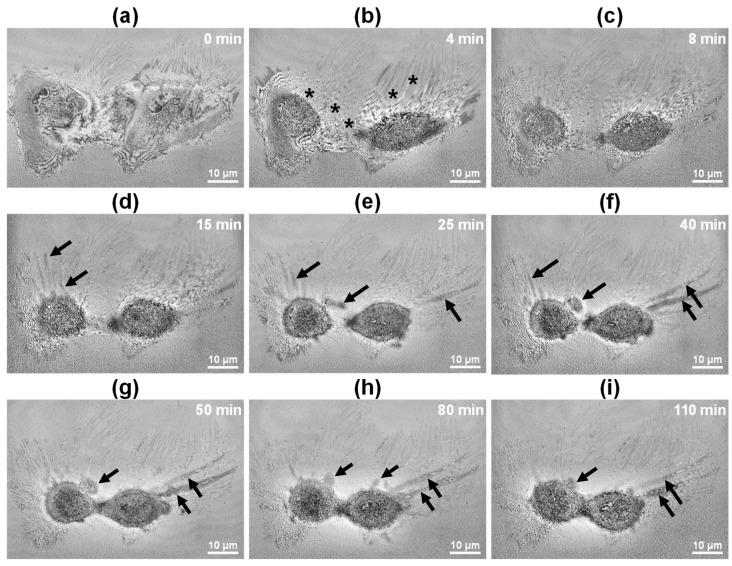
Time-lapse IRM-like FF-OCT imaging of ethanol-induced necrosis in HeLa cells. (**a**–**i**) *En face* IRM-like images from 0 to 110 min post-treatment, showing initial membrane discontinuity marked by asterisks at 4 min (**b**) and extracellular exudation indicated by arrows in panels (**d**–**i**). Progressive loss of cell–substrate adhesion and structural integrity is observed over time. Scale bar: 10 µm.

Figure 6 presents the dynamics at the cell–substrate interface of the two necrotic HeLa cells in Figure 5, as observed using IRM-like FF-OCT imaging. At 0 min, both cells were firmly attached to the culture substrate, maintaining stable adhesion structures (Figure 6a). However, starting at 4 min, the cell membranes exhibited rapid damage and loss of integrity (indicated by asterisks in Figure 6b), suggesting the rapid activation of the necrosis pathway. By 8 min, the cell–substrate boundary had become blurred (Figure 6c). Between 15 and 25 min, extracellular exudate spread was clearly visible (arrows in Figure 6d,e). These exudates exhibited low reflectivity in IRM-like images, likely due to light absorption and scattering by the cytoplasmic components. From 40 to 110 min, the cell body outline gradually disappeared, adhesion structures disassembled, and overall cell morphology collapsed (Figure 6f–i). This rapid membrane disruption and intracellular content leakage were markedly different from the gradual contraction and bleb formation observed during apoptosis. Compared with the IRM-like images of apoptotic cells in Figure 2, Figure 6 demonstrates a faster breakdown of cell boundaries, more extensive exudate release, and a fundamentally distinct pattern of morphological collapse. These findings indicate that IRM-like FF-OCT imaging enables effective qualitative classification of cell death pathways at the single-cell level, providing a real-time monitoring tool for necrosis progression.

## 4. Discussion

Our findings demonstrated that FF-OCT can effectively differentiate apoptotic and necrotic cell death at the single-cell level. By providing real-time, depth-resolved tomographic images, FF-OCT allowed for integrated morphological analysis of internal structures, surface topography, and cell–substrate interactions. Through multimodal imaging including an IRM-like mode, FF-OCT visualized dynamic processes such as membrane deformation, cytoskeletal remodeling, and cellular efflux. These observations aligned with known morphological markers of apoptosis and necrosis, supporting FF-OCT as a reliable and versatile imaging tool for cell death characterization.

Time-lapse FF-OCT images of apoptotic cells (Figure 2 and Figure 3) revealed morphological changes comparable to those observed in conventional fluorescence staining studies. Specifically, FF-OCT captured apoptotic hallmarks such as cellular volume reduction, surface echinoid spike formation, membrane blebbing, nuclear condensation, and increased optical density under doxorubicin treatment [32,33,34]. These findings suggest that key apoptotic features can be identified at high resolution without the need for staining or invasive treatment. In contrast, ethanol-induced necrosis (Figure 5 and Figure 6) exhibited rapid membrane disintegration, exudation of cellular contents, loss of cell–substrate adhesion, and extensive structural collapse [11,35,36,37], reflecting the pathological cascade of necrosis. The rapid destructive dynamics of necrosis were visualized through FF-OCT-based IRM-like imaging, highlighting the utility of FF-OCT in both quantitative and qualitative discrimination between apoptotic and necrotic pathways.

Apoptosis involves cytoskeletal reorganization driven by ATP depletion and caspase cascade activation, leading to actin filament contraction, cell shrinkage (apoptotic volume decrease), and membrane blebbing. FF-OCT-derived 3D topographic maps quantitatively illustrated localized surface protrusions and central elevation, while IRM-like imaging revealed progressive weakening of cell–substrate adhesion. In necrosis, factors such as ROS generation, membrane lipid bilayer damage, and calcium dysregulation induce rapid membrane rupture and leakage of intracellular contents. FF-OCT time-series images captured strong scattering signals and rapid exudation, while IRM-like images showed immediate disappearance of cell–substrate interference patterns, clearly illustrating the rapid structural collapse characteristic of necrosis. Overall, this multimodal approach effectively captures the contrasting dynamics of detachment during apoptosis and abrupt collapse during necrosis.

It is important to note that apoptosis and necrosis can present heterogeneous morphological characteristics depending on the cell type, microenvironment, and stimuli. For instance, some primary or stem cells may show atypical or mixed phenotypes under stress. The necrotic process can vary with factors such as membrane lipid composition or calcium influx [1,3,4,12]. Although this study focuses on HeLa cells as a representative cancer model, future applications of FF-OCT across diverse cell types, including stem cells and patient-derived cells, will further validate its generalizability.

However, the current platform has certain limitations. First, because signal intensity depends on RI contrast, it can be challenging to resolve intracellular regions with similar refractive indices—evident in the intermittent visualization of nuclear boundaries (Figure 1b and Figure 4a). Second, the current system’s scan rate (5 fps per FF-OCT image) limits its ability to capture rapid cell dynamics occurring within seconds. Third, unlike FF-OCT tomographic imaging, FF-OCT IRM-like imaging requires additional calibration, such as aligning the coherence gate to the substrate surface. If this is not properly calibrated, contrast degradation or image blurring may occur. Future improvements in hardware and postprocessing techniques are necessary to address these limitations and further enhance FF-OCT’s imaging performance. For example, dynamic FF-OCT (D-FF-OCT) technology [38] may be a promising solution to improve the visibility of internal structures. This leverages temporal fluctuations in interference signals caused by intrinsic organelle motility to enhance image contrast. These fluctuations enable visualization of intracellular dynamics and structures even in regions with low RI contrast. Moreover, single-shot FF-OCT technology [39] enables simultaneous acquisition of quadrature phase-shifted interference images on a single camera capture. A single tomogram can be reconstructed through simple arithmetic operations on these four images. Excluding computation time, the imaging speed is limited only by the camera frame rate, significantly enhancing temporal resolution and enabling the real-time observation of rapid cellular changes. For optimal IRM-like imaging, automated focus adjustment [40] using motorized stages and real-time monitoring of interference signal intensity at the culture dish surface may serve as an effective calibration method for enhancing imaging quality and reproducibility.

The FF-OCT-based analysis of cell death morphology holds promise for a variety of biomedical applications, including drug toxicity screening, analysis of anticancer drug mechanisms, biosensor-based cytotoxicity detection, and regenerative medicine. For instance, many anticancer drugs may induce apoptosis or necrosis depending on concentration and exposure duration. FF-OCT can differentiate these outcomes through morphological signatures, aiding in mechanism-of-action studies. In regenerative medicine, FF-OCT enables non-invasive 3D imaging of stem cell spheroids or engineered tissues, identifying early necrotic zones or apoptosis in 3D constructs without staining. This is crucial for evaluating product safety and function.

Integrating FF-OCT with machine learning algorithms may further support automated classification of cell states. Combining FF-OCT with complementary imaging modalities such as QPM or fluorescence microscopy could enhance the diagnostic accuracy and enable comprehensive cell viability assessment.

## 5. Conclusions

In summary, this study presented FF-OCT as a powerful, label-free imaging platform for distinguishing apoptosis and necrosis in single cells. By visualizing distinct dynamic features of apoptotic and necrotic processes, FF-OCT offers a promising approach for investigating cell viability and death mechanisms. Future developments in system speed, sensitivity, and automation may broaden its utility to complex biological models such as organoids.

## Data Availability

The data presented in this study will be made available on request.
